# Traditional herbal prescription LASAP-C inhibits melanin synthesis in B16F10 melanoma cells and zebrafish

**DOI:** 10.1186/s12906-016-1209-7

**Published:** 2016-07-16

**Authors:** Min Kyoung Kim, Chae Young Bang, Mi Yoon Kim, Jeung-Hoon Lee, Hyunju Ro, Min-Sun Choi, Dong-Il Kim, Young Pyo Jang, Se Young Choung

**Affiliations:** Department of Life and Nanopharmaceutical Sciences, College of Pharmacy, Kyung Hee University, Hoegi-dong, Dongdaemun-gu, Seoul, 130-701 South Korea; Division of Pharmacognosy, College of Pharmacy, Kyung Hee University, Hoegi-dong, Dongdaemun-gu, Seoul, 130-701 South Korea; Department of Preventive Pharmacy and Toxicology, College of Pharmacy, Kyung Hee University, Hoegi-dong, Dongdaemun-gu, Seoul, 130-701 South Korea; Department of Dermatology, School of Medicine, Chungnam National University, 266 Munhwa-ro, Jung-gu, Daejeon, 301-747 South Korea; Department of Biological Science, College of Bioscience and Biotechnology, Chungnam National University, 88 Daekak-ro, Yuseong-gu, Daejeon, 305-764 South Korea; Dongguk University Bundang Oriental Hospital, 268 Buljeong-ro, Bundang-gu, Seongnam-si, Gyeonggi-do South Korea; Dongguk University International Hospital, 27 Donkguk-ro, Ilsandong-gu, Goyang-si, Gyeonggi-do South Korea

**Keywords:** LASAP-C, Herbal prescription, Melanin, Tyrosinase, TRP-1, TRP-2, Zebrafish

## Abstract

**Background:**

In this study, the anti-melanogenesis efficacy of clinically used herbal prescription LASAP-C, which consists of four herbal medicines—Rehmanniae Radix Crudus, Lycii Fructus, Scutellariae Radix, and Angelicae Dahuricae Radix, was investigated.

**Methods:**

The chemical profile of LASAP-C was established by conducting ultra-performance liquid chromatography-electrospray ionization-mass spectrometry. Anti-melanogenic efficacy was evaluated by tyrosinase, tyrosinase-related protein (TRP)-1, and TRP-2 expression in B16F10 melanoma cells. In vivo evaluation was performed by using zebrafish model.

**Results:**

Molecular evidences suggested that melanin synthesis was inhibited via the down-regulation of tyrosinase, tyrosinase-related protein (TRP)-1, and TRP-2 expression in B16F10 melanoma cells treated with LASAP-C. The anti-melanogenesis efficacy was also confirmed in vivo by using the zebrafish model.

**Conclusion:**

The results of this study provide strong evidences that LASAP-C can be used as an active component in cosmeceutical products for reducing excess pigmentation in the human skin.

## Background

Melanin, the major pigment of human skin, is secreted by melanocytes in the basal layer of the epidermis. It is known to be overproduced in the cases of chronic sun exposure, melasma, or other hyperpigmentation diseases [1, 2]. Melanin biosynthesis can be inhibited by avoiding ultraviolet (UV) exposure, preventing melanocyte metabolism and proliferation, and blocking activities of enzymes such as tyrosinase [3]. Among the various causes of excessive melanin synthesis, UV radiation has been known to induce the formation of reactive oxygen species (ROS) in the skin, such as singlet oxygen and superoxide anion, promoting biological damage in exposed tissues via iron-catalyzed oxidative reactions [4–6]. These ROS enhance melanin biosynthesis and DNA damage and might induce melanocyte proliferation [7–9].

Tyrosinase, a copper-containing monooxygenase, is a key enzyme that catalyzes two major reactions—hydroxylation of tyrosine and oxidation of l-dopa—of melanin synthesis by melanocytes [10]. Dopa oxidation produces a highly reactive intermediate that is further oxidized to form melanin via a free radical-coupling pathway [10].

In this study, we evaluated the anti-melanogenesis effect of the traditional herbal prescription LASAP-C, which was found to be effective in an in vitro tyrosinase assay. LASAP-C was derived from Korean traditional herbal prescriptions and utilized as skin tone brightening agent in local Clinique [11]. LASAP-C is composed of four herbal medicines Rehmanniae Radix Crudus, Lycii Fructus, Scutellariae Radix, and Angelicae Dahuricae Radix. We used B16F10 melanoma cells as a model system to investigate the effects of LASAP-C on anti-melanogenesis and evaluated the underlying molecular mechanisms such as the effect on the expression of tyrosinase, tyrosinase-related protein (TRP)-1, and TRP-2. We also determined the inhibitory effect of LASAP-C on melanin synthesis by using an in vivo model of zebrafish.

## Methods

### Preparation of LASAP-C water extract

The raw material used for preparing LASAP-C (235 g) was *Rehmannia glutinosa* Libosch. var. *purpurea* Makino (Scrophulariaceae; root, 100 g; voucher specimen number: DUMCKM2015-040), *Lycium chinense* Mill. (Solanaceae; fruit, 50 g; voucher specimen number: DUMCKM2015-008), *Scutellaria baicalensis* Georgi (Labiatae; root, 50 g; voucher specimen number: DUMCKM2015-081), and *Angelica dahurica* Bentham et Hooker f. (Umbelliferae; root, 35 g; voucher specimen number: DUMCKM2015-031). The herbal medicines were Korea Food and Drug Administration-certified and purchased from a local herbal market in South Korea; their botanical authenticity was confirmed by Prof. Dong Il Kim. A voucher specimen has been deposited at the Herbarium of the College of Korean Medicine, Dong-guk University, Ilsan, Korea. LASAP-C was extracted with 1 L distilled water at 100 °C for 4 h by using a Soxhlet extractor [12]. The extract was filtered through a filter paper (Hyundai Micro Co., Ltd., Korea), and the filtrate was freeze-dried (yield, 62 g) and maintained at 4 °C.

### Chemicals and reagents

High-purity nitrogen gas was purchased from Shinyang Oxygen Co. (Seoul, South Korea). High-performance liquid chromatography-grade acetonitrile and acetic acid were purchased from Duksan Pure Chemicals Co. (Ansan, South Korea). 1-phenyl-2-thiourea (PTU) was purchased from Sigma (ST Louis, MO, USA) for the zebrafish study. Dulbecco’s modified Eagle’s medium (DMEM; SH30243.01), fetal bovine serum (FBS; SH30396.03), and penicillin-streptomysin solution (SV30010) were purchased from Hyclone Laboratories Inc. (Logan, UT, USA). Dimethyl sulfoxide (DMSO; D2650), kojic acid (K-3125), l-dopa (37830), and synthetic melanin (M-8631) were purchased from Sigma (St. Louis, MO, USA). All chemicals and reagents were of analytical grade.

The protease inhibitor cocktail Complete™ was purchased from Roche (Mannheim, Germany). Protein assay reagent (#500-0006) was purchased from Bio-Rad (Richmond, CA, USA). Tyrosinase (M-19, sc-7834), TRP-1 (M-19, sc-10448), TRP-2 (D-18, sc-10451), *β*-actin (I-19, sc-1616) antibodies, and commercially available secondary antibody were purchased from Santa Cruz Biotechnology, Inc. (Dallas, TX, USA).

### Ultra performance liquid chromatography tandem mass spectrometry analysis

Before being injected to ultra performance liquid chromatography (UPLC) system, the LASAP-C extract was dissolved in distilled water to yield a 20 mg/mL concentration and filtered through 0.2 μm syringe filter (Millipore, Bedford, MA, USA). UPLC-diode array detection analysis was performed using Waters ACQUITY Ultra Performance LC system equipped with Sample Manager-FTN, quaternary solvent manager, and photodiode array detector (Waters, Milford, MA, USA). The analysis was performed using ACQUITY UPLC HSS C18 column (50 × 2.1 mm i.d.; 1.8 μm). The UV detector monitoring wavelength was set to 300 nm. The mobile phase consisted of acetonitrile and water acidified with 0.1 % acetic acid (solvents A and B, respectively). The gradient program was as follows: 0 min, 1 % of solvent A; 2 min, 1 % of solvent A; 6 min, 20 % of solvent A; 8 min, 20 % of solvent A; 20 min, 35 % of solvent A; 22 min, 100 % of solvent A; 23 min, 1 % of solvent A; 25 min, 1 % of solvent A, at a flow rate of 0.7 mL per minute. The injection volume was 0.5 μL.

AccuTOF® single-reflectron time-of-flight mass spectrometer equipped with an electrospray ionization (ESI) source (JEOL, Peabody, MA, USA) was used for UPLC-mass spectrometry (MS) study and was operated using Mass Center system version 1.3.7b (JEOL, Peabody, MA, USA). In the positive ion mode, the values were as follows: orifice 1 = 80 V, ring lens = 10 V, and orifice 2 = 5 V. The ion guide potential and detector voltage were set to 2000 V and 2300 V, respectively. ESI parameters were set as follows: needle electrode = 2000 V, nitrogen gas flow rate as nebulizer = 1 L/min, nitrogen gas flow rate as desolvating agent = 3 L/min, desolvating chamber temperature = 250 °C, and orifice 1 temperature = 80 °C. Mass scale calibration was performed using YOKUDELNA calibration kit (JEOL, Tokyo, Japan) for accurate mass measurements and elemental composition calculations. MS acquisition range was set from *m/z* 150 to 2500.

### B16F10 melanoma cell culture

B16F10 melanoma cells derived from C57BL/6 J mouse were purchased from ATCC (Manassas, VA, USA). Cells were incubated in DMEM supplemented with 10 % FBS, 100 U/mL penicillin, and 100 mg/mL streptomycin at 37 °C under 5 % CO_2._

### Cell viability assay

The safety of LASAP-C was determined by checking cell viability after treatment with various concentrations (0, 10, 50, 100, 200, and 400 μg/mL) of LASAP-C by using 3-[4, 5-dimethylthiazol-2-yl]-2, 5 diphenyl tetrazolium bromide (MTT) colorimetric assay. This assay measures the metabolic reduction of MTT to formazan (blue) by mitochondrial dehydrogenase, which is active only in living cells [13]. B16F10 melanoma cells were incubated in 24-well plates at a density of 10^4^ cells/well for 24 h. On the second day, cells were exposed to various concentrations of LASAP-C water extract for 48 h. The media was then removed; the cells were washed with phosphate-buffered saline (PBS, pH 7.4) and grown in 0.5 mg/mL MTT, which was prepared in PBS, at 37 °C. After 4 h, the MTT reagent was removed, formazan crystals were dissolved in DMSO solution, and absorption values were read at 540 nm by using an enzyme-linked immunosorbent assay (ELISA) microplate reader (Bio-Tek Instruments Inc., Winooski, VT, USA).

### Measurement of cellular tyrosinase activity

B16F10 melanoma cells (5 × 10^4^ cells/well) were seeded in a 6-well culture plate and treated with various concentrations (10, 50, and 100 μg/mL) of LASAP-C for 48 h. They were washed with ice-cold PBS and lysed with phosphate buffer (pH 6.8) containing 1 % Triton X-100. They were then disrupted by freezing and thawing, and whole cell lysates were clarified by centrifugation at 10,000 *g* for 5 min. After protein levels were quantified and protein concentrations were adjusted using lysis buffer, 90 μL of each lysate, containing the same amounts of protein, was placed in each well of a 96-well plate, and 10 μL of 10 mM l-DOPA was added. Control wells contained 90 μL of lysis buffer and 10 μL of 10 mM l-DOPA. After incubation at 37 °C for 15 min, the dopachrome was observed by measuring the absorbance at 475 nm by using an ELISA reader.

### Melanin content measurement in B16F10 melanoma cells

The inhibitory effect of LASAP-C on the synthesis of melanin in B16F10 melanoma cells was evaluated by measuring melanin content in cells that were treated with three concentrations (10, 50, and 100 μg/mL) of LASAP-C for 48 h. The cells were dissolved in 0.2 mL of 1 N NaOH and boiled at 100 °C for 30 min. The amount of melanin was determined by measuring absorbance at 460 nm by using the ELISA microplate reader and normalizing with protein level of cell lysates.

### Western blot analysis

The effects of LASAP-C on the expression of tyrosinase, TRP-1, and TRP-2 were investigated using western blot analysis. The total amount of protein content of each supernatant was measured using Bradford assay (Bio-Rad, Richmond, CA, USA). B16F10 melanoma cells were lysed in cell lysis buffer (62.5 mM Tris–HCl (pH 6.8), 2 % sodium dodecyl sulfate (SDS), 5 % *β*-mercaptoethanol, protease inhibitor (Complete™, Roche, Mannheim, Germany), 2 mM phenylmethylsulfonyl fluoride, 10 mM EDTA, 50 mM NaF, and 1 mM Na_3_VO_4_). Next, 10 μg of protein per lane was separated by 8 % SDS-polyacrylamide gel electrophoresis and blotted onto polyvinylidene fluoride membranes, which were then saturated with 5 % non-fat dried milk in Tris-buffered saline containing 0.1 % Tween 20. Blots were exposed to specific primary antibodies at a dilution of 1:1000 overnight at 4 °C, followed by incubation with a horseradish peroxidase-conjugated secondary antibody for 2 h at room temperature. The blots were developed using an enhanced chemiluminescence detection system (Thermo FisherScientific Inc., Rockford, IL, USA).

### Measurement of the effect of LASAP-C on pigmentation in zebrafish embryos

The effect of LASAP-C on the pigmentation of zebrafish embryos was determined according to the method described previously [14]. Adult zebrafish (wild-type, AB) were obtained from a commercial dealer, and 10–15 fish were maintained in a 5 L acrylic tank under the following conditions: 28.5 °C, with a 14/10 h light/dark cycle. Zebrafish were fed three times a day and six days a week with Tetramin flake food (Tetra, Melle, Germany) supplemented with live brine shrimps (*Artemia salina*). Embryos were obtained from natural spawning, which was induced in the morning by turning on the light. The embryos were collected within 30 min. Synchronized embryos were placed in 96-well plates containing 200 μL embryo medium (13.7 mM NaCl, 5.4 mM KCl, 25 μM Na_2_HPO_4_, 44.1 μM KH_2_PO_4_, 1.3 mM CaCl_2_, 1 mM MgSO_4_ · 7H_2_O, and 4.17 mM NaHCO_3_, ca. 10 drops 1 M NaOH to adjust pH 7.2). LASAP-C (10 and 100 μg/mL in 0.1 % DMSO) was added to the embryo medium from 9 h post fertilization (hpf) to 72 hpf (63 h exposure). Three embryos were assigned for each well. Further, 0.2 mM 1-phenyl-2-thiourea (PTU) was used to generate transparent zebrafish without interfering the developmental process [15]; these zebrafish were considered as a standard positive control. The effect on the pigmentation of zebrafish was observed under a stereomicroscope MZ16 (Leica Microsystems, Ernst-Leitz-Strasse, Germany). For observation, embryos were dechorionated using forceps, anesthetized in tricaine methane sulfonate solution (Sigma, St Louis, MO, USA), mounted in 3 % methyl cellulose on a depression slide (Aquatic EcoSystem, Apopka, FL, USA), and photographed using stereomicroscope MZ16.

### Statistical analysis

Results are presented as means ± standard error of the mean of at least three independent experiments. Statistical analysis was performed using *t*-test. Student’s *t*-test and paired *t*-test were performed to assess the differences between the means. *P* values less than 0.05 were considered statistically significant.

## Results and discussion

### Establishment of the UPLC profile of LASAP-C extract

**Fig. 1:**
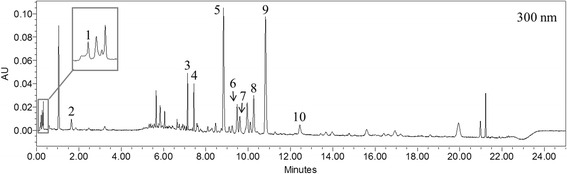
Ultra-performance liquid chromatogram of LASAP-C

The standard chromatogram for LASAP-C was established by using the UPLC system. The representative chromatogram of LASAP-C is shown in Fig. 1. Since several peaks were present as major components, other smaller peaks appeared close to the baseline. The major peaks of the chromatogram were identified by performing UPLC-ESI-MS.

### Identification of phytochemicals by UPLC-ESI-MS

The retention time, observed mass, mass difference with theoretical mass, and proposed compounds of ten peaks are listed in Table 1. The ten phytochemicals in LASAP-C were as follows: betaine from Lycii Fructus; catalpol from Rehmanniae Radix Crudus; aviprin and byakangelicin from Angelicae dahuricae Radix; chrysin-6-*C*-arabinose-8-*C*-glucose, chrysin-6-*C*-glucose-8-*C*-arabinose, baicalin, wogonin-7-*O*-glucuronide, oroxylin A-7-*O*-glucuronide, and baicalenin from Scutellariae Radix. These ten compounds were identified based on the UV and MS spectra of previously reproted. Although Rehmanniae Radix Crudus was the major component of LASAP-C in terms of quantity, phytochemicals from Scutellariae Radix were prominent as major peaks in the UPLC-MS study. The raw material of Rehmanniae Radix Crudus contained upto 77 % of water contents depends on cultivars [16] and many primary metabolites such as saccharides, vitamins, and amino acids [17, 18]. The most common iridoidal glycoside of raw Rehmanniae Radix Crudus is catalpol and its content in the dried root has been reported as 0.142 to 0.225 % [16]. The high moisture content and a low catalpol content may lead to the representation as a minor peak in the UPLC-MS study.

**Table 1:** The observed and calculated mass numbers of UPLC peaks of LASAP-C

Peak No.	RT (min)	Theoretical mass [M + H]^+^	Observed mass [M + H]^+^	Mass difference (mmu)	Fragment ions	Identification	Reference
1	0.21	118.08680	118.08978	2.98	-	betaine	[31]
2	1.65	362.12129	362.12673	5.44	-	catalpol	[18]
3	7.14	549.16081	549.15920	−8.96	531.15137/434.18988	chrysin-6-*C*-arabinose-8-*C*-glucose	[32]
4	7.44	549.16081	549.15912	−9.04	531.15132/434.18978	chrysin-6-*C*-glucose-8-*C*-arabinose	[32]
5	8.84	447.09272	447.08914	−3.58	271.06377	baicalin	[32]
6	9.48	305.10195	305.10251	0.56	203.03895	aviprin	[33]
7	9.56	335.11251	335.11512	2.61	233.05536	byakangelicin	[33]
8	10.26	461.10782	461.10570	−2.12	285.07676	wogonin-7-*O*-glucuronide	[32]
9	10.82	461.10782	461.10585	−1.97	285.07221	oroxylin A-7-*O*-glucuronide	[32]
10	12.45	271.06065	271.06377	3.12	-	baicalenin	[32]

Acteoside extracted from Rehmanniae Radix Crudus was reported to inhibit melanogenesis in B16F10 cells by the extracellular signal-regulated kinase (ERK) pathway [19] even though it was not detected in LASA-C total extract but valuable evidence of the skin whitening expectation on this herbal source. Aviprin and byakangelicin from Angelicae dahuricae Radix was identified as major peaks and byakangelicin was reported to inhibit melanogenesis without any influence on cell proliferation but aviprin has been reported to increase melanin production, in contrast [20]. However, Angelicae dahuricae Radix has been reported as a valuable skin whitening agent evidenced by the suppression of tyrosinase synthesis and inhibition of melanogenesis [20, 21]. It may be attributable to its various kinds of coumarin compounds which were reported to have different effects on skin whitening. For example, aviprin (oxygenated linear-prenylfurocoumarin) exhibited weak stimulation of melanin production but phellopterin (linear-prenylfurocoumarin), heraclenin (oxygenated linear-prenylfurocoumarin), and columbianadin (acylated angular-dihydrofurocoumarin) suppressed melanogenesis [20]. Baicalin from Scutellariae Radix has been known to restrain melanogenesis of human melanocytes induced by ultraviolet [22].

### Effects of LASAP-C on tyrosinase activity and melanin synthesis in B16F10 cells

The cytotoxicity of LASAP-C was determined by treating B16F10 cells with LASAP-C (0, 10, 50, 100, 200, and 400 μg/mL) for 2 days and measuring the cell viability (data not shown). The LASAP-C extract did not induce any cytotoxicity up to 100 μg/mL concentration, but dose-dependently reduced viability at higher doses (53 % at 200 μg/mL, 68 % at 300 μg/mL, and 70 % at 400 μg/mL). According to the cell viability results, cells were treated with LASAP-C at a maximum concentration of 100 μg/mL.

**Fig. 2:**
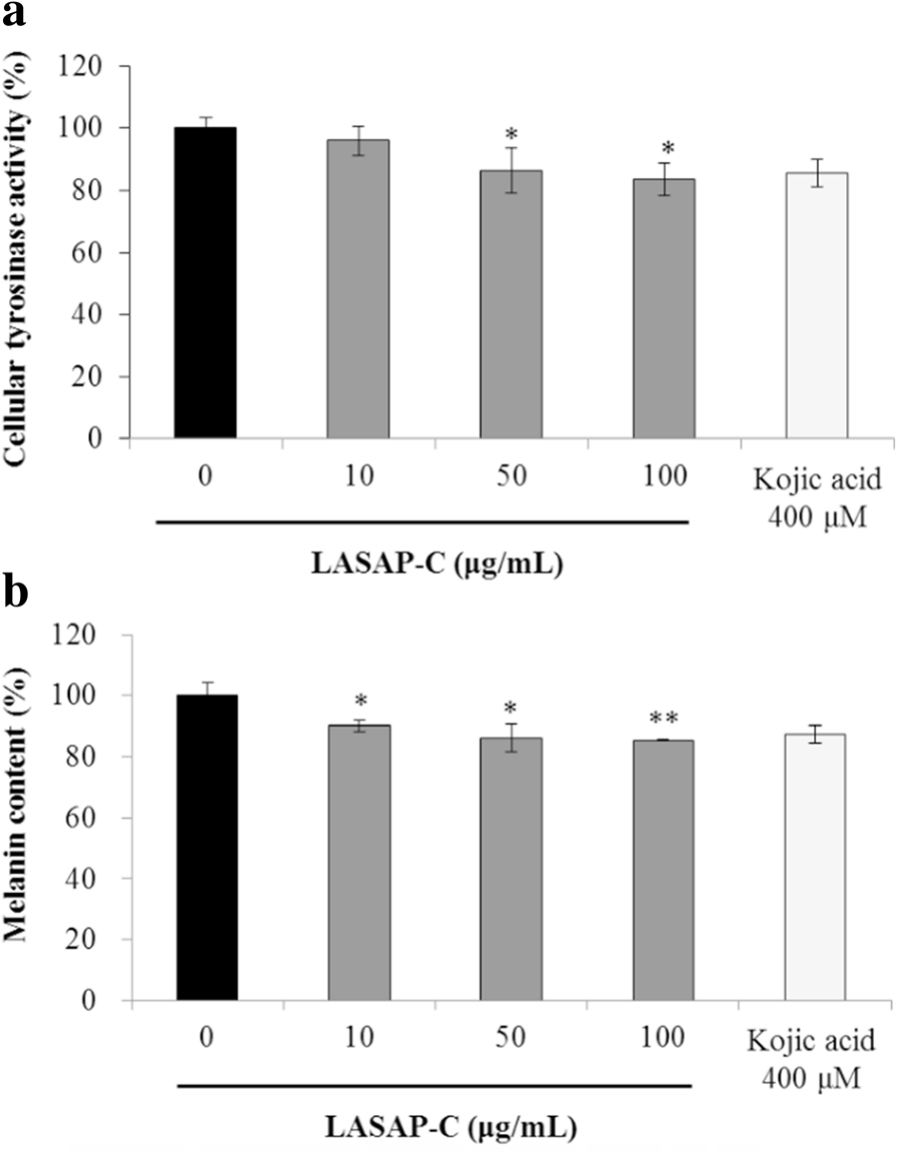
Inhibitory effects of LASAP-C on cellular tyrosinase activity (**a**) and melanin production (**b**). Each measurement was performed in B16F10 melanoma cells in triplicate; the data represent mean ± standard error ^*^ *p* < 0.05 and ^**^ *p* < 0.01 vs. without LASAP-C

Tyrosinase is a rate-limiting enzyme in melanin synthesis. The biosynthesis of melanin is initiated by the catalytic oxidation of tyrosine to dopa by tyrosinase in the presence of dopa as a cofactor [23]. Tyrosinase activity of cell lysates treated with various concentrations (10, 50, and 100 μg/mL) of LASAP-C was measured; dose-dependent reduction in tyrosinase activity was observed compared to that of the blank and positive control (400 μM kojic acid). The B16F10 cells retained 96.0, 86.02, and 83.7 % tyrosinase activity after treatment with 10, 50, and 100 μg/mL LASAP-Cs (Fig. 2a). LASAP-C reduced cellular melanin content in B16F10 melanoma cells compared to that in the positive control (Fig. 2b). Cells cultured with 10, 50, and 100 μg/mL of LASAP-C showed significantly decreased levels of melanin (Fig. 2b). B16F10 cells retained 90.18, 86.02, and 85.53 % melanin content after treatment with 10, 50, and 100 μg/mL LASAP-C.

### Effects on the expression of tyrosinase, TRP-1, and TRP-2 (dopachrome tautomerase) proteins

**Fig. 3:**
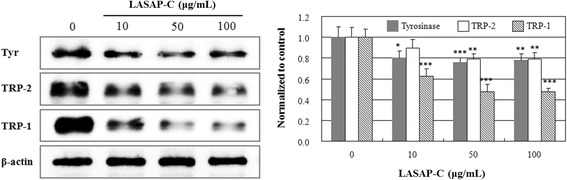
The effects of LASAP-C extract on protein levels of melanin synthesis enzymes. The activities of tyrosinase, tyrosinase-related protein (TRP)-1, and TRP-2 in B16F10 melanoma cells were measured. Each determination was made in triplicate; the data represent mean ± standard error. ^*^ *p* < 0.05, ^**^ *p* < 0.01, and ^***^ *p* < 0.001 vs. without LASAP-C

Melanin is synthesized within melanosomes that contain three major pigment enzymes: tyrosinase, TRP-1, and TRP-2 [24–26]. The protein levels of tyrosinase, TRP-1, and TRP-2 in B16F10 cells were dose-dependently down-regulated by treatment with LASAP-C extract (Fig. 3). The anti-melanogenesis activity of LASAP-C was shown to be directly related with the down-regulation of the major genes associated with the key enzymes involved in melanogenesis.

### Effects of LASAP-C on melanin synthesis in zebrafish

**Fig. 4:**
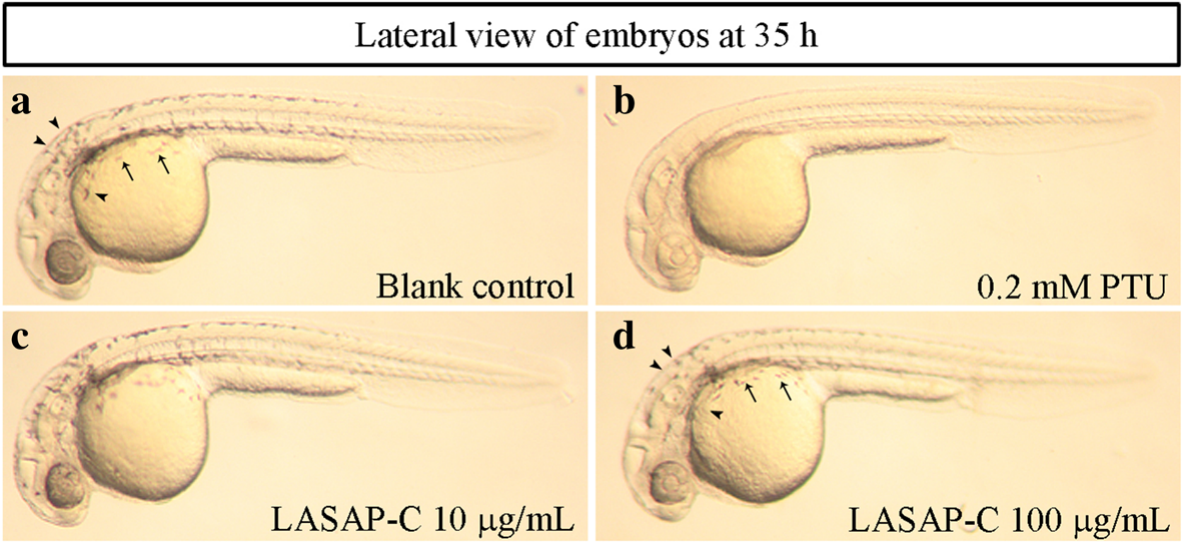
The effects of LASAP-C extract on melanogenesis in zebrafish. Synchronized embryos were treated with 10 and 100 μg/mL LASAP-C. The effects on zebrafish pigmentation were observed using a stereomicroscope. In blank control and 100 μg/mL LACAP-C treated zebrafish, the pigmentation was pointed as black arrowhead and shrinkage of pigment cells was marked as black arrow. Lateral view of embryos at 31 h post fertilization: **a** blank control, **b** 0.2 mM 1-phenyl-2-thiourea (PTU), **c** 10 μg/mL LASAP-C, and **d** 100 μg/mL

Recently, the zebrafish model is being actively used in various in vivo assays as a good alternative system to rodents [27–29]. The effects on the pigmentation of zebrafish were determined stereomicroscopically. PTU, a well-known tyrosinase inhibitor, was used as a positive control of pigment production inhibition in zebrafish [30]. LASAP-C extract dose-dependently inhibited melanin synthesis in zebrafish embryos (Fig. 4c and d). The potency of the anti-melanogenic effect of LASAP-C (100 μg/mL) was mild compare to the potency of 0.2 mM PTU, the positive control. LASAP-C treatment not only reduced pigmentation (black arrowhead) but also caused shrinkage of pigment cells (black arrow) in Fig. 4.

## Conclusion

In this study, the anti-melanogenic effect of LASAP-C was confirmed, and five major components of this traditional prescription were identified by UPLC-ESI-MS for quality control for further research and development.

The anti-melanogenic effect of LASAP-C extract was thought to occur via the inhibition of three key enzymes involved in melanin synthesis. The dose-dependent inhibitory effect of LASAP-C on tyrosinase, TRP-1, and TRP-2 expression indicated that this herbal prescription was directly related with the inactivation of genes encoding these proteins.

The whitening effect of LASAP-C was also validated using zebrafish as a whole-animal model for phenotype-based screening of LASAP-C extract. As expected, LASAP-C extract remarkably inhibited body pigmentation in zebrafish embryos, reconfirming its anti-melanogenic efficacy. Taken together, these results suggest that LASAP-C has a strong potential to be used as an active ingredient in various cosmeceutical products for skin whitening.

## Abbreviations

DMSO, dimethyl sulfoxide; l-DOPA, l-3,4-dihydroxyphenylalanine; PTU, 1-phenyl-2-thiourea; ROS, reactive oxygen species; TRP-1, tyrosinase-related protein-1; TRP-2, tyrosinase-related protein-2; UV, ultraviolet
